# Molecular characteristics and clinical outcomes of *EGFR* exon 19 indel subtypes to EGFR TKIs in NSCLC patients

**DOI:** 10.18632/oncotarget.22768

**Published:** 2017-11-30

**Authors:** Jian Su, Wenzhao Zhong, Xuchao Zhang, Ying Huang, Honghong Yan, Jinji Yang, Zhongyi Dong, Zhi Xie, Qing Zhou, Xiaosui Huang, Danxia Lu, Wenqing Yan, Yi-Long Wu

**Affiliations:** ^1^ Guangdong Lung Cancer Institute, Guangdong Provincial Key Laboratory of Translational Medicine in Lung Cancer, Medical Research Center of Guangdong General Hospital & Guangdong Academy of Medical Sciences, Guangzhou 510080, China; ^2^ Guangdong Lung Cancer Institute, Guangdong General Hospital & Guangdong Academy of Medical Sciences, Guangzhou 510080, China

**Keywords:** non-small cell lung cancer, *EGFR* mutation, EGFR-TKI, *EGFR* exon 19 deletion, *EGFR* exon 19 insertion

## Abstract

Patients with non-small cell lung cancer (NSCLC) with activating epidermal growth factor receptor *(EGFR)* mutations (exon 19 deletions and L858R) benefit from EGFR tyrosine kinase inhibitors (TKIs). However, some researchers have reported that responses to TKIs differ by subtypes of *EGFR* exon 19 mutations. We retrospectively analyzed *EGFR* exon 19 deletion subtypes and their correlation with clinical outcomes of treatment with TKIs. A cohort of 2664 consecutive patients with NSCLC was enrolled. A total of 440 *EGFR* exon 19 deletions were defined as 39 subtypes. Among them, 158 patients with advanced lung adenocarcinoma with *EGFR* exon 19 deletion mutations received EGFR-TKIs. There were no significant differences in progression-free survival or overall survival among patients with non-LRE deletions, delE746, or delL747 (*P* = 0.463 and *P* = 0.464, respectively). Furthermore, two patients with *EGFR* exon19 insertion had durable response to EGFR-TKIs. In conclusion, *EGFR* exon 19 is highly fragile, resulting in many different deletion and insertion subtypes. There were no significant differences in clinical outcomes after TKI treatment across the different subtypes. It is necessary to attempt to identify all patients with exon 19 deletions so that they can be offered TKI treatment.

## INTRODUCTION

Epidermal growth factor receptor (EGFR) tyrosine kinase inhibitors (TKIs) have become standard therapy for advanced non-small cell lung cancer (NSCLC). Patients with activating *EGFR* mutations benefit from EGFR-TKIs such as erlotinib and gefitinib, which prolong progression-free survival (PFS) and improve the response rate [1–3]. The most frequent *EGFR* mutations with sensitivity to TKIs in NSCLC are exon 19 deletions and the single-point substitution L858R in exon 21, which account for about 44% and 41% of all *EGFR* mutations, respectively, and are termed common mutations [4]. Additional rare *EGFR* mutations that have been identified include G719X in exon 18 (about 4%) and L861Q in exon 21 (2%), which are modestly sensitive to EGFR-TKIs, and insertions in exon 20 (about 4%), which are less sensitive to EGFR-TKIs [5–7].

Among *EGFR* mutations, deletions of exon 19 are more complex because they consist of different subtypes. The majority of cases encompass the amino acids from codons L747 to E749 (designated as the LRE fragment) [4]. According to the Catalogue for Somatic Mutations in Cancer (COSMIC) database for *EGFR,* the most frequent exon deletions are delE746-A750 (68.9%), followed by delL747-P753insS (6.0%), delL747-T751 (4.1%), and delL747-A750insP (3.9%) [8]. Previous researchers [9] showed that different subtypes of *EGFR* exon 19 are associated with different clinical outcomes in response to first-line TKI therapy, with TKIs showing better efficacy for delE746 than delL747. Therefore, deletion locations may affect TKI efficacy [10].

*EGFR* genotyping is now routine practice in the management of NSCLC. Different methods have been developed to identify *EGFR* mutations. Sanger sequencing is the standard assessment method for *EGFR* mutation identification, but it is time-consuming and lacks sensitivity. Various polymerase chain reaction (PCR) methods, such as amplification refractory mutation system (ARMS) and peptide nucleic acid (PNA) clamping, have been developed to detect *EGFR* mutations with increased sensitivity and in less time. However, the ARMS approach cannot cover all types of *EGFR* exon 19 deletions, and a false negative result is sometimes obtained [11, 12].

In this study, we retrospectively analyzed the molecular changes of *EGFR* exon 19 and their associations with clinical outcomes of treatment with TKIs. Based on the different base pair changes in exon 19, we also aimed to develop a sensitive approach to detect all of the subtypes of *EGFR* exon 19 deletions.

## RESULTS

### Characteristics of patients with *EGFR* exon 19 deletions

Among the 2664 specimens, 896 (33.6%) harbored at least one *EGFR* mutation, among which 440 (49.1%) were exon 19 deletions and 368 (41.1%) were exon 21 L858R mutations, 20 (2.2%) G719X, 9 (1.0%) L861Q, and 42 (4.7%) exon 20 insertions. In addition, 4 exon 19 insertions were found (0.4%). Characteristics of the patients with *EGFR* exon 19 deletions are summarized in Table 1. The median patient age was 57 years (range, 22–86 years). Most patients with exon 19 deletions were nonsmokers (73.1%), and had adenocarcinomas (96.8%).

**Table 1:** Characteristics of NSCLC patients with *EGFR* exon19 deletion

Characteristics	N (%)	No. of patients (%)			*P*
		E746	L747	non-LRE	
cases^*^	432	332	91	9	
**Median age** years (range)	57(22-86)	57(22-86)	54(33-85)	60(38-66)	0.226
**Sex**					1.000
male	202(46.8)	155(46.7)	43(47.3)	4(44.4)	
female	230(53.2)	177(53.3)	48(52.7)	5(55.6)	
**Smoking status**^#^					0.882
smoker	115(26.6)	87(26.3)	25(27.5)	3(33.3)	
non-smoker	316(73.1)	244(73.8)	66(72.5)	6(66.7)	
**Histology**					0.401
AC	418(96.8)	323(97.3)	86(94.5)	9(100)	
SCC	14(3.2)	9(2.7)	5(5.5)	0	

AC: adenocarcinoma, SCC:squamous cell carcinom, NSCLC: non-small cell lung cancer, EGFR: epidermal growth factor receptor, ^*^Of the 440 samples, 8 could not be typed.

^#^ 1 patient with unknown smoking status.

### Molecular characteristics of EGFR exon 19 deletions

**Figure 1:**
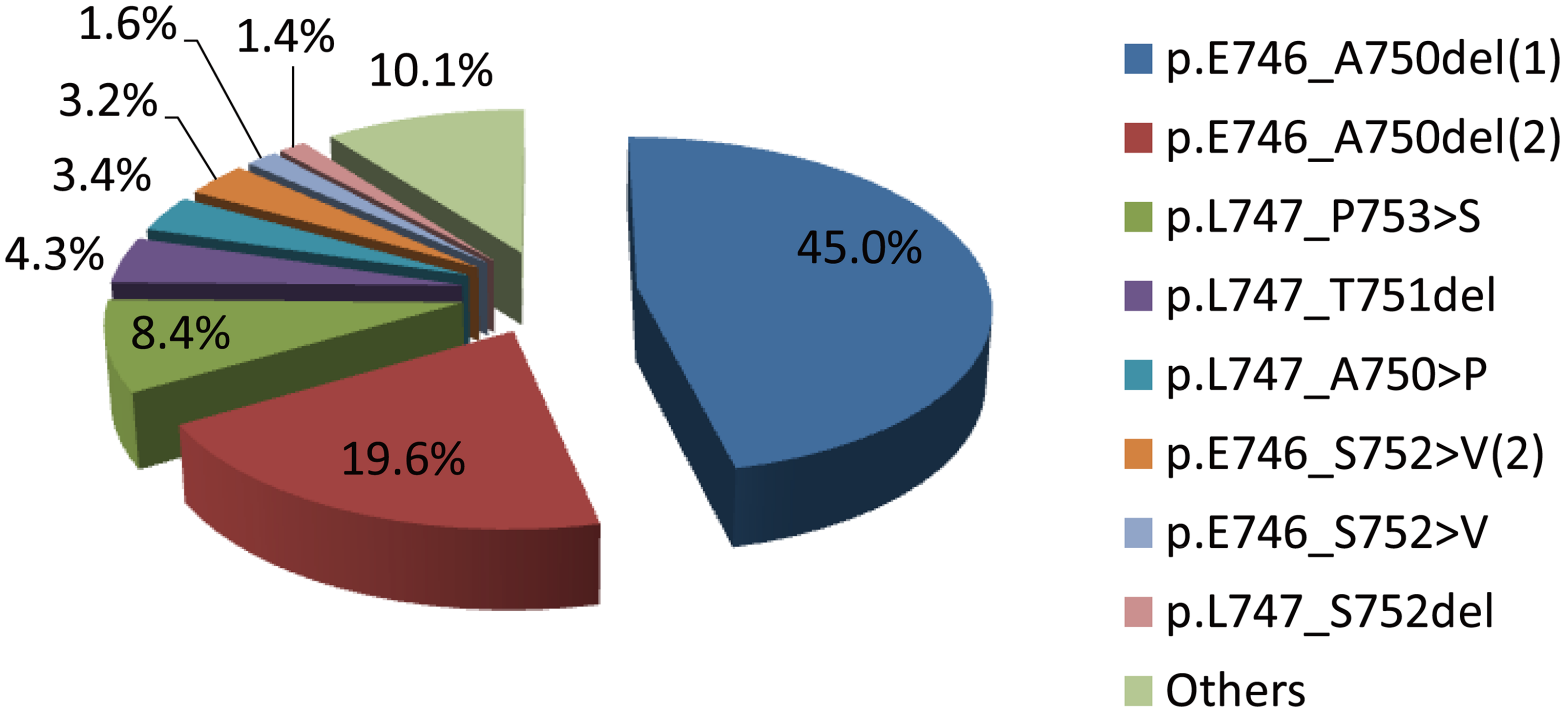
Frequency of *EGFR* exon19 deletion subtypes (N=440)

Among the 440 samples of exon 19 deletions, we defined 39 exon 19 deletion subtypes. The most frequent subtypes were p.E746_A750del (64.6%), p.L747_P753>S (8.4%), p.L747_T751del (4.3%), p.L747_A750>P (3.4%), p.E746-S752>V(2) (3.2%), p.E746_S752>V (1.6%), and p.L747_S752del (1.4%) (Table 2, Figure 1). As shown in Table 2, the base pairs of all of the subtypes deleted encompassed a wide range from 2235 to 2281, while deleted amino acids ranged from E746 to D761. More than half of deletion subtypes (61.1%) were accompanied by base pair insertions.

**Table 2:** *EGFR* exon 19 deletions subtypes in patients with NSCLC

			Nucleotide sequence (2230-2262)				
No.	Amino acid change	Base pair change	atc aag gaa tta aga gaa gca aca tct ccg aaa gcc aac aag gaa atc ctc gat gaa gcc	Cases	%	Cosmic ID	Missed^*^
			I K E L R E A T S P K A N K E I L D E A				
**1**	**p.E746_A750del(1)**	**c.2235_2249del15**	atc aag gaa tta aga gaa gca aca tct ccg aaa gcc aac aag gaa atc ctc gat gaa gcc	198	**45.0**	6223	
**2**	p.E746_T751>KV	c.2235_2252>ggt	atc aag gaa tta aga gaa gca acGGTa tct ccg aaa gcc aac aag gaa atc ctc gat gaa gcc	1	0.2	NA	1
**3**	p.E746_A750>HS	c.2236_2248>ctaa	atc aag gaa tta aga gaa gCAT Tca aca tct ccg aaa gcc aac aag gaa atc ctc gat gaa gcc	1	0.2	NA	1
**4**	p.E746_A750del(3)	c.2236_2249del14	atc aag gaa tta aga gaa gca aca tct ccg aaa gcc aac aag gaa atc ctc gat gaa gcc	3	0.7	NA	3
**5**	**p.E746_A750del(2)**	**c.2236_2250del15**	atc aag gaa tta aga gaa gca aca tct ccg aaa gcc aac aag gaa atc ctc gat gaa gcc	86	**19.6**	6225	
**6**	p.E746_T750del(4)	c.2236_2251>a	atc aaggaa tta aga gaa gca aAca tct ccg aaa gcc aac aag gaa atc ctc gat gaa gcc	1	0.2	NA	1
**7**	p.E746_T751>FPT	c.2236_2251>tttccaa	atc aag gaa tta aga gaa gca aTTTCCAAca tct ccg aaa gcc aac aag gaa atc ctc gat gaa gcc	1	0.2	NA	1
**8**	p.E746_T751>L	c.2236_2252>ct	atc aag gaa tta aga gaa gca acCTa tct ccg aaa gcc aac aag gaa atc ctc gat gaa gcc	1	0.2	51502	1
**9**	p.E746_S752>IP	c.2236_2255>atacc	atc aag gaa tta aga gaa gca aca tcA TAC Ct ccg aaa gcc aac aag gaa atc ctc gat gaa gcc	1	0.2	NA	1
**10**	p.E746_P753>MS	c.2236_2257>atgt	atc aag gaa tta aga gaa gca aca tct cATGTcg aaa gcc aac aag gaa atc ctc gat gaa gcc	1	0.2	NA	1
**11**	p.E746_P753>MS	c.2236_2257>atgtc	atc aag gaa tta aga gaa gca aca tct cATGTCcg aaa gcc aac aag gaa atc ctc gat gaa gcc	1	0.2	NA	1
**12**	**p.E746_T751>A**	**c.2237_2251del15**	atc aag gaa tta aga gaa gca aca tct ccg aaa gcc aac aag gaa atc ctc gat gaa gcc	3	0.7	12678	
**13**	p.E746_T751>APT	c.2237_2253>caccaact	atc aag gaa tta aga gaa gca aca CACCAACTtct ccg aaa gcc aac aag gaa atc ctc gat gaa gcc	1	0.2	NA	1
**14**	p.E746_T751>VA	c.2237_2253>ttgct	atc aag gaa tta aga gaa gca acaTT GCT tct ccg aaa gcc aac aag gaa atc ctc gat gaa gcc	3	0.7	12416	3
**15**	**p.E746_S752>V**	**c.2237_2255>t**	atc aag gaa tta aga gaa gca aca tcTt ccg aaa gcc aac aag gaa atc ctc gat gaa gcc	7	**1.6**	12384	
**16**	p.E746_P753>VS	c.2237_2257>tct	atc aag gaa tta aga gaa gca aca tct cTCTcg aaa gcc aac aag gaa atc ctc gat gaa gcc	3	0.7	18427	3
**17**	p.E746_S752>V(2)	c.2237_2257>ttc	atc aag gaa tta aga gaa gca aca tct cTTCcg aaa gcc aac aag gaa atc ctc gat gaa gcc	14	**3.2**	NA	14
**18**	p.E746_K754>GG	c.2237_2261>tcgg	atc aag gaa tta aga gaa gca aca tct ccg aaTCGGa gcc aac aag gaa atc ctc gat gaa gcc	1	0.2	NA	1
**19**	p.E746_E749del	c.2238_2247del10	atc aag gaatta aga gaa gca aca tct ccg aaa gcc aac aag gaa atc ctc gat gaa gcc	2	0.5	NA	2
**20**	p.E746_E749>P	c.2238_2249>gcc	atc aag gaa tta aga gaa gc GCCa aca tct ccg aaa gcc aac aag gaa atc ctc gat gaa gcc	3	0.9	NA	3
**21**	**p.L747_E749del**	**c.2239_2247del9**	atc aag gaa tta aga gaa gca aca tct ccg aaa gcc aac aag gaa atc ctc gat gaa gcc	2	0.5	6218	
**22**	p.L747_A750>P	c.2239_2250>cca	atc aag gaa tta aga gaa gcaCCA aca tct ccg aaa gcc aac aag gaa atc ctc gat gaa gcc	15	**3.4**	133195	16
**23**	p.L747_T751>P	c.2239_2253>cca	atc aag gaa tta aga gaa gca aca CCA tctccg aaa gcc aac aag gaa atc ctc gat gaa gcc	5	1.1	51527	4
**24**	**p.L747_T751>N**	**c.2239_2253>aat**	atc aag gaa tta aga gaa gca aca AAT tct ccg aaa gcc aac aag gaa atc ctc gat	1	0.2	51503	
**25**	p.L747_S752>PT	c.2239_2254>ccga	atc aag gaa tta aga gaa gca aca tCCGAct ccg aaa gcc aac aag gaa atc ctc gat gaa gcc	1	0.2	NA	1
**26**	p.L747_P753>NS	c.2239_2256>aattcg	atc aag gaa tta aga gaa gca aca tct ccg AATTCGaaa gcc aac aag gaa atc ctc gat gaa gcc	1	0.2	NA	1
**27**	p.L747_S752>PI	c.2239_2256>caaata	atc aag gaa tta aga gaa gca aca tctCAAATAccg aaa	1	0.2	NA	1
**28**	**p.L747_S752del**	**c.2239_2256del18**	atc aag gaa tta aga gaa gca aca tct ccg aaa gcc aac aag gaa atc ctc gat gaa gcc	6	**1.4**	6255	
**29**	p.L747_P753>S	c.2239_2259>tc	atc aag gaa tta aga gaa gca aca tct ccg TCaaagcc aac aag	1	0.2	NA	1
**30**	p.L747_K754del	c.2239_2262del24	atc aag gaa tta aga gaa gca aca tct ccg aaagcc aac aag gaa atc ctc gat gaa gcc	1	0.2	24970	1
**31**	**p.L747_T751>S**	**c.2240_2251del12**	atc aag gaa tta aga gaa gca aca tct ccg aaa	1	0.2	6210	
**32**	**p.L747_T751del**	**c.2240_2254del15**	atc aag gaa tta aga gaa gca aca tct ccg aaa gcc aac aag gaa atc ctc gat gaa gcc	19	**4.3**	12369	
**33**	**p.L747_P753>S**	**c.2240_2257del18**	atc aag gaa tta aga gaa gca aca tct ccg aaa gcc aac aag gaa atc ctc gat gaa gcc	37	**8.4**	12370	
**34**	p.A750_I759>PT	c.2248_2276>ccaac	atc aag gaa tta aga gaa gca aca tct ccg aaa gcc aac aag gaa atCCAACc ctc gat gaa gcc	1	0.2	5023004	1
**35**	p.T751_I759>T	c.2253_2275del23	atc aag gaa tta aga gaa gca aca tct ccg aaa gcc aac aag gaa atc ctc gat gaa gcc	1	0.2	NA	1
**36**	p.S752_I759del(2)	c.2253_2276del24	atc aag gaa tta aga gaa gca aca tct ccg aaa gcc aac aag gaa atc ctc gat gaa gcc	2	0.5	13556	2
**37**	p.T751_I759>N	c.2252_2276>a	atc aag gaa tta aga gaa gca aca tct ccg aaa gcc aac aag gaa atAc ctc gat gaa gcc	1	0.2	96856	1
**38**	p.T751_D761>NLY	c.2252_2281>atctct	atc aag gaa tta aga gaa gca aca tct ccg aaa gcc aac aag gaa atc ctc gATCTCTat gaa gcc	1	0.2	NA	1
**39**	p.S752_I759del	c.2254_2277del24	atc aag gaa tta aga gaa gca aca tct ccg aaa gcc aac aag gaa atc ctc gat gaa gcc	3	0.7	6255	3
**40**	untyped			8	1.8		
				440			72

^*^The subtypes excluded by a popular *EGFR* mutation detection commercial kit; Blue upper letters represent insert base pairs. Red lower letters represent delete base pairs. Orange lower letters represent the position of the PNA probe.

### Sensitivity of ddPCR method based on PNA clamping

**Figure 2:**
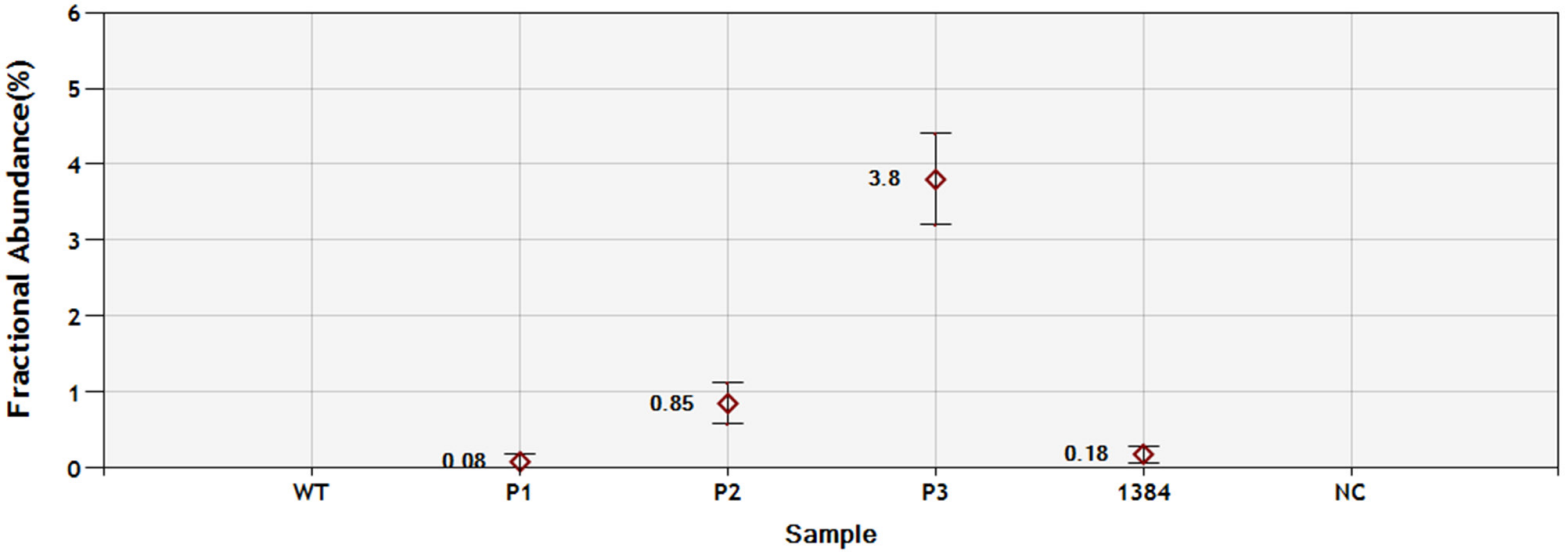
Sensitivity of ddPCR with PNA clamping for *EGFR* exon19 deletion detection P1: 0.1% Multiplex I cfDNA Reference Standard (HD780); P2: 1% Multiplex I cfDNA Reference Standard (HD780); P2: 5% Multiplex I cfDNA Reference Standard (HD780); 1384: patient sample; NC: negative control.

Of the 93 *EGFR* exon 19 deletion samples in 2015, 91 could be confirmed by ddPCR and Sanger sequencing. One sample was observed to be wild type by Sanger sequencing and deletion by ddPCR. Another sample, which was non-LRE subtype, could be confirmed by Sanger sequencing but not by ddPCR (Supplementary Table 1). Utilizing the Multiplex I cfDNA Reference Standard, the sensitivity of the ddPCR method was 0.08% (Figure 2).

### Response to TKIs

**Figure 3:**
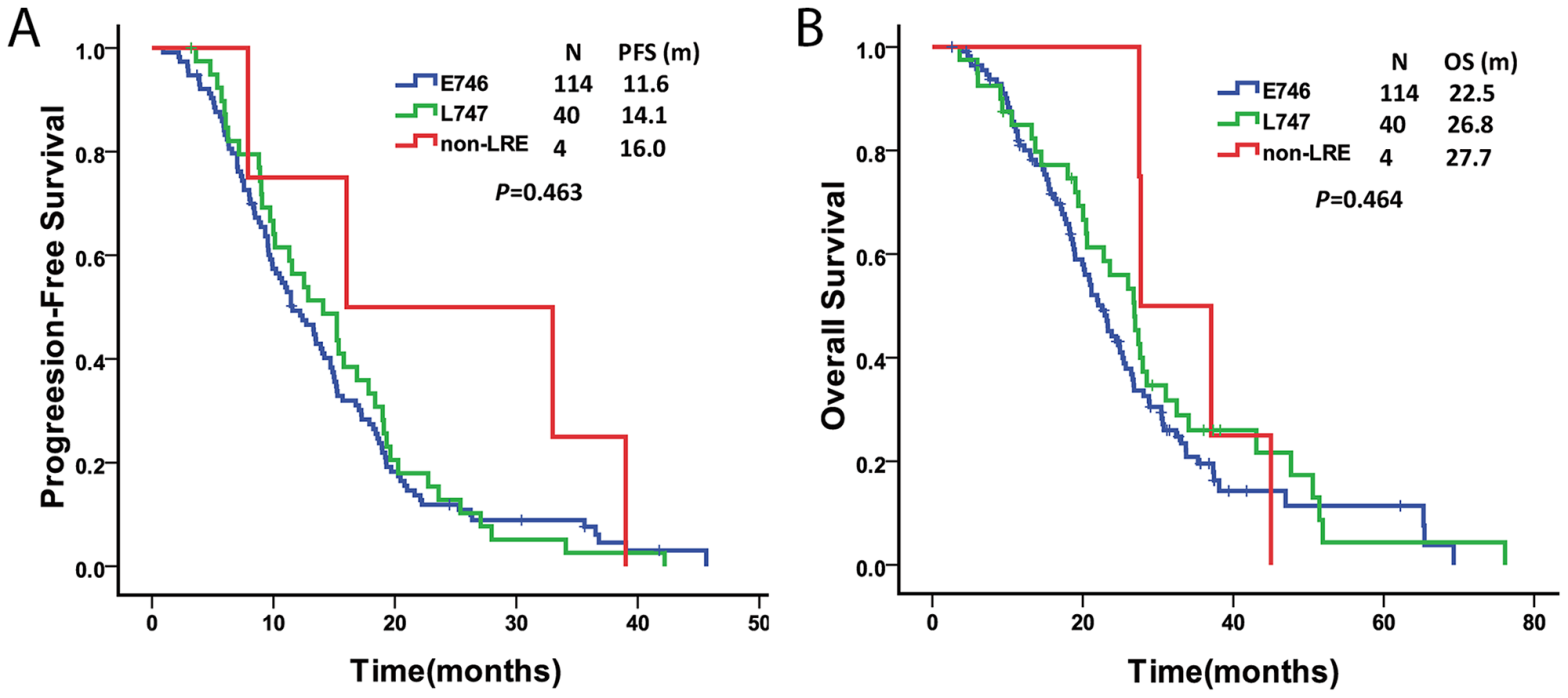
Comparison of progression-free survival **(A)** and overall survival **(B)** among Del-E746, Del-L747 and non-LRE groups.

A total of 158 patients with *EGFR* exon19 deletions were treated with TKIs (gefitinib or erlotinib); the characteristics of these patients are shown in Supplementary Table 2. The deletion subtype was classified into three groups according to the first codon of the exon 19 deletion. There were 114 samples (72.2%) that had deletions starting at E746 (E746-group), 40 samples (25.3%) with deletions starting at L747 (L747-group), and 4 samples (2.5%) with deletions starting at T751 or S752, which did not include the LRE amino acid (non-LRE group). The response rate to TKIs was 69.2% (74/107) in the E746-group, 83.3% (30/36) in the L747-group, and 75.0% (3/4) in the non-LRE group (*P* = 0.198). Patients with non-LRE deletions had a relatively long median PFS compared to those with deletions from E746 or L747, but the difference was not significant (16.0, 11.6, and 14.1 months, respectively; *P* = 0.463). The OS was not different among the E746, L747, and non-LRE groups (*P* = 0.464) (Figure 3).

Multivariate analysis of factors associated with PFS and OS in 158 patients with advanced lung adecocarcinoma treated with EGFR-TKIs was performed and is summarized in Supplementary Table 3. Eastern Cooperative Oncology Group (ECOG) performance status was shown to be independent predictors of PFS.

**Figure 4:**
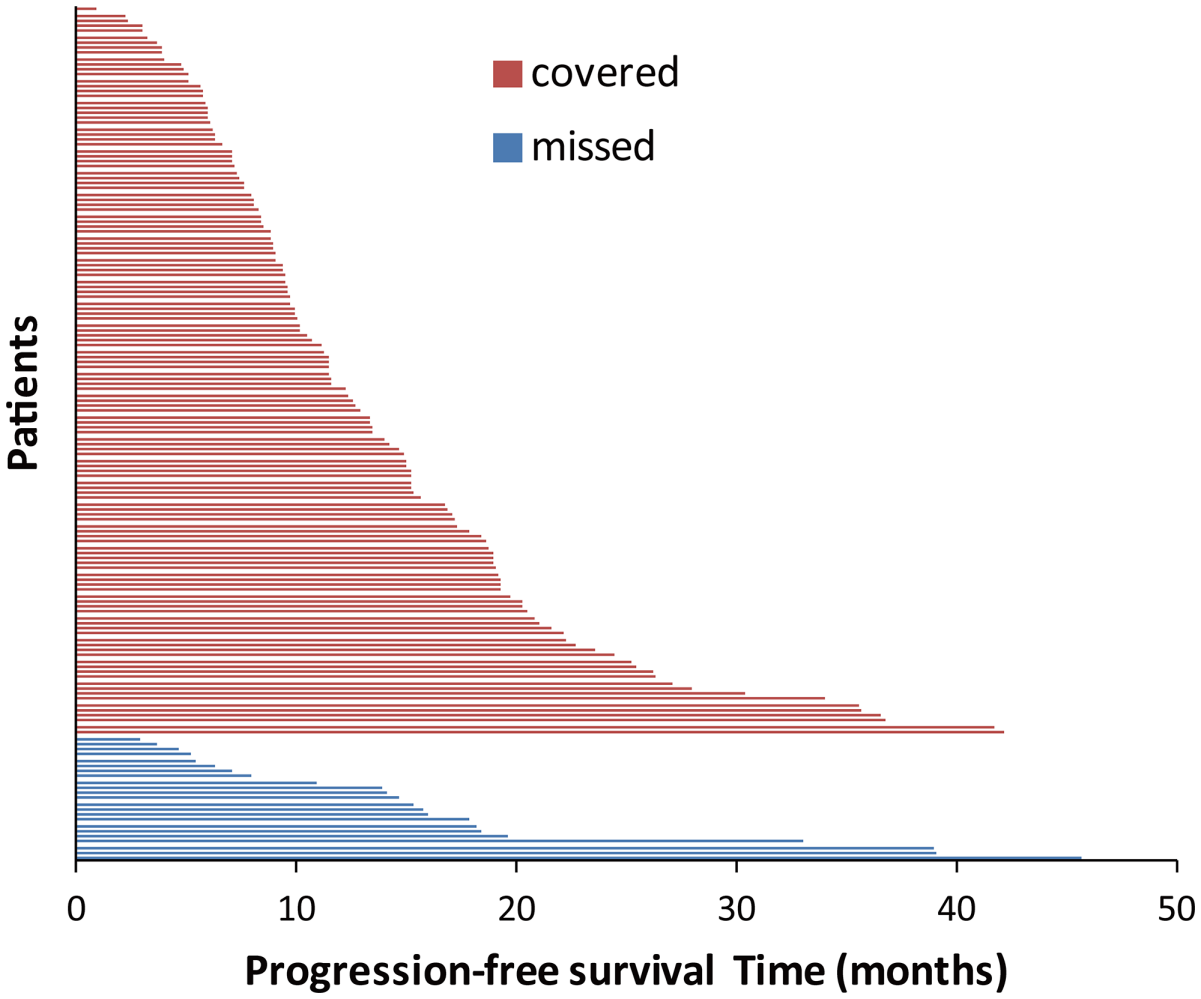
Progression-free survival according to different EGFR exon 19 subtypes Covered group represents the *EGFR* deletion subtypes that the popular commercial kit includes, while missed group represents *EGFR* deletion subtypes which the popular commercial kit excludes.

Among the 158 patients, 135 had *EGFR* exon 19 deletion subtypes that could be detected with ARMS (Covered group), while 23 could not (Missed group). There was no significant difference in median PFS between the Covered and Missed groups (12.2 vs. 14.7 months, *P* = 0.430). Figure 4 shows the details of PFS according to the different subtypes of exon 19 deletion in the Covered group and the Missed group.

## DISCUSSION

The present article retrospectively analyzed *EGF*R mutation data from a cohort of 2664 consecutive patients with NSCLC. A total of 440 (16.5%) samples had exon 19 deletions that could be further classified into 39 subtypes. Among them, eight subtypes were predominant and accounted for 86.9%, consistent with the COSMIC database for *EGFR* and the public literature [8]. Of the 39 subtypes, 20 have been reported in the COSMIC database, but 19 subtypes had not.

In China, ARMS, available as a commercial kit, is one of most widely used methods for detecting *EGFR* mutations. However, because it is based on an allelic-specific PCR technique, this method can only detect a few exon 19 deletion subtypes [12–14]. In our data, only 10 subtypes were covered by the commercial kit (shown in Table 2 with purple letters). That is, 29 subtypes could not be detected using this kind of commercial kit (Table 2). Thus, 72 (16.4%, 72/440) deletion samples would have been missed and the affected patients would lose the opportunity to receive TKI targeted treatment.

To our knowledge, the present study constituted the first analysis of *EGFR* exon 19 deletion subtypes in Chinese patients with NSCLC. Our data showed that deletions occur throughout almost the entire exon 19 amino acid string from E746 to D761 involving 16 amino acids, and could be defined as 39 subtypes. Over half of the subtypes were complex, with an accompanying insertion. Marchetti et al. showed with next generation sequencing that 20% of *EGFR* exon 19 deletions are complex frame shift deletions producing a net in-frame change. In some cases, even Sanger sequencing could not define the exact sequence of the mutant allele. The data of these authors support the hypothesis that a region within exon 19 is particularly fragile and preferentially susceptible to microdeletions [14]. We have found 19 new deletion subtypes that have not been reported in the COSMIC database. These results also support the idea that *EGFR* exon 19 is very fragile. Thus, it is very difficult to detect all exon 19 mutations in the clinical practice.

A few researchers have reported differences in sensitivity to EGFR-TKIs among patients with different exon 19 deletion subtypes, but the conclusions have been controversial. Chung et al. reported that patients with non-LRE deletions have a relatively short median PFS that is not significantly different from that of those with deletions E746 or L747 (5.9, 9.8, and 10.5 months, respectively; *P* = 0.662) [10]. Lee et al. [9] and Kaneda et al. [15] reported that patients with E746 had longer PFS than those with L747, and the difference was significant. However, Sutiman et al. [16] have just shown that there were no significant differences in PFS and OS between the L747 and E746 groups. On the other hand, with a new grouping method, the shortest OS was observed in the 15n-deletion “non-ELREA” group (*P* = 0.025). Our data, however, are contrary to the findings of previous studies in that patients with non-LRE deletions had a relatively long median PFS, but it was not significantly different from that of those with deletions E746 or L747 (16.0, 11.6, and 14.1 months, respectively; *P* = 0.463). No differences were observed in OS among the three groups (*P* = 0.464). The small size of the non-LRE group may partly account for the inconsistency. Furthermore, applying the grouping method used by Sutiman et al. [16], we also could not observe any difference in PFS and OS across the five patient groups (data not shown). Nonetheless, the basic explanations remain unclear. Therefore, it is necessary to attempt to identify all patients with exon 19 deletions so that they can be offered TKI treatment.

In addition to *EGFR* exon 19 deletions, the presence of *EGFR* exon 19 insertion subtypes that have shown sensitivity to EGFR-TKIs has also been reported [17–19]. In the present study, we found 4 patients with exon 19 insertions; these patients were all women, nonsmokers, and had adenocarcinomas (Table 3). The sequence analysis showed an 18 nucleotide duplication sequence inserted at position c.2231–2235 in exon 19, adjacent to the deletion hot spot site. The hot spot duplication sequence was PVAI. Two out of the 4 patients were treated with TKIs. P1 received erlotinib as first-line therapy and showed a partial response; her time to progression (TTP) of disease was 15.5 months. P2 received gefitinib as first-line therapy and had stable disease, and her TTP was 24 months. Although *EGFR* exon 19 insertions have rarely been documented, the frequency was 0.35% in a Hong Kong cohort and about 0.26% in non-Asian patients [17, 18]. Previous studies have showed a striking correlation between TKI sensitivity and *EGFR* insertion mutations both *in vitro* and in clinical studies [17–19].

**Table 3:** Cases with *EGFR* exon 19 insertions

ID	Amino acid change	Base pair change	Sex	Age,year	Histology	Smoking status	TKI	Best response	TTP,m
1	p.E746_L747ins**VPVAIK**	c.2236_2227dupTTCCCGTCGCTATCAAGG	female	60	AC	never	Erlotinib	PR	15.5
2	p.I744_K745ins**KIPVAI**	c.2231_2232dupTAAAATTCCCGTCGCTAT	female	37	AC	never	Gefitinib	SD	24
3	p.I744_K745ins**KIPVAI**	c.2231_2232dupTAAAATTCCCGTCGCTAT	female	69	AC	never	NA	NA	NA
4	p.K745_E746ins**IPVAIK**	c.2234_2235dupAATTCCCGTCGCTATCAA	female	78	AC	never	NA	NA	NA

AC: adenocarcinoma, PR: partial response, SD: stable disease, NA: not available, TTP: time to progression.

Overall, *EGFR* exon 19 alterations are very complex, including different deletion subtypes and insertions. Patients with these alterations could benefit from EGFR-TKIs. It is important to identify a robust method that can identify all of the exon 19 alterations. In China, ARMS is routinely used to detect *EGFR* mutation. With the limitations of that method, only a few exon 19 deletion subtypes can be identified. PNA clamping is one approach to detect *EGFR* mutations with high sensitivity and time savings [12, 14, 20, 21]. A PNA, or peptide nucleic acid, is an artificially synthesized DNA analog that binds strongly to its complementary DNA sequence. While the specifically designed PNA probe inhibits PCR amplification in wild-type sequences, it allows greater amplification of mutant type sequences. The advantage of PNA-PCR compared to ARMS is that it can detect a larger number of subtypes of *EGFR* exon 19 deletions. Furthermore, the cost of PAN-PCR is affordable. Because the deleted base pairs of exon 19 encompass a wide range of positions within different subtypes, the choice of sequence for a PNA probe is important to provide coverage of more exon 19 deletion subtypes. The present study showed that the deleted base pairs of all of the subtypes encompassed a position from 2235 to 2281. We chose the “AGAGAAGCAACATCT” sequence for the PNA probe because it could cover all of the known subtypes of exon 19 deletions. In this study, we found that one of the 93 exon 19 deletions, which is a non-LRE subtype, could not be detected using PNA-PCR. Further analysis showed that the reverse PCR primer was located on the deletion site. Another PCR set should be designed to detect non-LRE deletion subtypes. Combining the PNA clamping approach with a ddPCR system could improve the detection sensitivity to 0.08%.

Recently, next generation sequencing (NGS) was introduced into clinical analysis for detecting oncogenic mutations including *EGFR* mutations. Previous studies have shown that NGS is a potent method that can identify unknown mutations with high sensitivity. However, NGS consists of a number of steps, including template preparation, sequencing and imaging, and data analysis. It is a labor-intensive and time-consuming method requiring specialized and costly facilities, restricting its use in routine practice [14, 22, 23].

There are several limitations to the present study. First, this is a retrospective study with a small sample size, and therefore there may be bias in the assessment of PFS. Second, the TKI treatment cohort is relatively small. As a result, only four patients with non-LRE exon 19 deletion types were treated with TKIs.

In conclusion, *EGFR* exon 19 is highly fragile, resulting in many different deletion and insertion subtypes. There were no significant differences in clinical outcomes after TKI treatment across the different subtypes. It is necessary to attempt to identify all patients with exon 19 deletions so that they can be offered TKI treatment.

## MATERIALS AND METHODS

### Patients

A cohort of 2664 consecutive patients with NSCLC was enrolled in this retrospective study. *EGFR* mutations in exons 18–21 were detected clinically by Sanger sequencing between 2010 to 2014 and by fragment analysis and the SNaPshot method in 2015 at Gungdong Lung Cancer Institute (GLCI). A total of 93 specimens with *EGFR* exon 19 deletions identified by fragment analysis in 2015 underwent further analysis of exon 19 by PNA clamping and Sanger sequencing.

TKI treatment was administered to a total of 158 patients with advanced lung adenocarcinoma with *EGFR* exon 19 deletion mutations which were identified with Sanger sequencing. Patients were excluded from further analysis if they received TKIs as neoadjuvant therapy or had lung squamous cell carcinoma. Survival was compared among different subtypes of exon 19 *EGFR* mutations in these 158 patients. Patients’ clinical data were extracted from electronic medical records at GLCI. Chest computed tomography was performed every 8–12 weeks as a routine clinical procedure to confirm patient response and evaluate disease progression. Tumor response was assessed according to Response Evaluation Criteria in Solid Tumors (RECIST) guidelines, version 1.1. The study was approved by the ethics committee of Guangdong General Hospital (GDREC2013185h(R2)). Informed consent was obtained from each patient.

### Detection of *EGFR* mutations by Sanger sequencing

*EGFR* mutations were detected by Sanger sequencing using a previously described protocol [24]. Briefly, PCR was performed to amplify exons 18–21 of *EGFR*. PCR was performed in a 25-μL volume containing 20 ng genomic DNA, 12.5 μL of Premix EXTaq HotStart version (Takara Bio Inc., Shiga, Japan), 5 μmol of each primer, and 3 μL of nuclease-free water. Then, 10 μL of the PCR products were purified with exonuclease I and alkaline phosphatase (shrimp) (Takara Bio Inc.). The purified products were sequenced bidirectionally with BigDye Terminator v3.1 (Applied Biosystems, Foster City, CA, USA) and an ABI 3730 Genetic Analyzer (Applied Biosystems) according to the manufacturer’s protocol. The sequencing data were analyzed using Sequencing Analysis Software v5.2 (Applied Biosystems).

### Detection of *EGFR* exon 19 deletions by fragment analysis

Deletional mutations in exon 19 of *EGFR* were detected using fragment analysis as described previously [24]. Briefly, PCR was performed using a FAM-labeled primer, and the resultant amplicon was separated using capillary electrophoresis and then analyzed in an ABI 3730 Genetic Analyzer (Applied Biosystems) according to the manufacturer’s protocol.

### Detection of *EGFR* exon 19 deletions by ddPCR with PNA clamping

A total of 93 *EGFR* exon 19 deletions identified by fragment analysis in routine practice in 2015 were subjected to PNA clamping with droplet digital PCR (ddPCR) and Sanger sequencing to confirm exon 19 deletion. PNA clamping with ddPCR was carried out using a Bio-Rad QX200 droplet system (Bio-Rad Laboratories, Hercules, CA, USA) according to the manufacturer’s protocol. The primers and probes were identical to those previously reported [25], but a modified EX19_PNA probe was used. The sequence of the EX19_PNA probe was: 5’-AGAGAAGCAACATCT-3’, which targets the common exon 19 deletion region and could cover more subtypes of exon 19 deletions than the primer previously used, according to our clinical data.

Briefly, the PCR amplification system was as follows: 10 μL of 2× digital PCR supermix for probes (Bio-Rad Laboratories), 1.8 μL of 10 μM exon19 forward and reverse primers mix, 1.8 μL of 10 μM exon 2 forward and reverse primers mix, 1.8 μL of 10 μM EX19_PNA probe, 1 μL of 10 μM exon 19 probe, 1 μL of 10 μM exon 2 probe, 1 μL of 20 ng/μL DNA, and water added to 20 μL. Multiplex I cfDNA Reference Standard (HD780, Horizon Discovery, Cambridge, UK) was used to assay the sensitivity for detecting *EGFR* exon 19 deletions at 5%, 1%, 0.1%, and 0.1% allelic frequencies.

### Statistics

Overall survival (OS) and PFS were compared among different patient groups using the Kaplan-Meier method with log-rank tests. Multivariate analyses for OS and PFS were conducted using the Cox proportional hazards model. Associations between mutations and clinical and biological characteristics were analyzed by the χ2 or Fisher’s exact test. All data were analyzed using the Statistical Package for the Social Sciences Version 17.0 Software (SPSS Inc., Chicago, IL, USA). The two-sided significance level was set at *P* < 0.05.

## SUPPLEMENTARY MATERIALS TABLES



## Abbreviations

ARMS: amplification refractory mutation system
COSMIC: Catalogue for Somatic Mutations in Cancer
ddPCR: droplet digital polymerase chain reaction
EGFR: epidermal growth factor receptor
NGS: next generation sequencing
NSCLC: non-small cell lung cancer
OS: overall survival
PFS: progression-free survival
PNA: peptide nucleic acid
RECIST: Response Evaluation Criteria in Solid Tumors
TKI: tyrosine kinase inhibitor
TTP: time to progression
