# Supplementary material for: Molecular characteristics and clinical outcomes of EGFR exon 19 indel subtypes to EGFR TKIs in NSCLC patients
Source: Oncotarget. 2017 Nov 30;8(67):111246–57. doi: 10.18632/oncotarget.22768 (PMC5762318; doi:10.18632/oncotarget.22768)
Supplement: Supplementary file 1 [file oncotarget-08-111246-s001.pdf]

Molecular characteristics and clinical outcomes of *EGFR* exon 19 indel subtypes to EGFR TKIs in NSCLC patients

SUPPLEMENTARY MATERIALS

Supplementary Table 1: *EGFR* exon19 deletion subtypes in 2015

ID	Amino acid change	Base pair change	cases
1	p.E746_A750del(1)	c.2235_2249del15	40
2	p.E746_R750>AF	c.2236_2248>ctaa	1
3	p.E746_A750del(2)	c.2236_2250del15	23
4	p.E746_P753>MS	c.2236_2257>atgt	1
5	p.E746_T751>VA	c.2237_2253>ttgct	1
6	p.E746_S752>V	c.2237_2255>t	2
7	p.E746_P753>VS	c.2237_2257>tct	1
8	p.E746_S752>V(2)	c.2237_2257>ttc	1
9	p.E746_E749>P	c.2238_2249>gcc	1
10	p.L747_A750>P	c.2239_2250>cca	5
11	p.L747_T751>N	c.2239_2253>aat	1
12	p.L747_S752>SD	c.2239_2254>ccga	1
13	p.L747_S752del	c.2239_2256del18	1
14	p.L747_E749 del	c.2239-2247del9	1
15	p.L747_T751del	c.2240_2254del15	4
16	p.L747_P753>S	c.2240_2257del18	5
17	p.S752_I759del	c.2253-2276del24	1
18	untyped		3
Total			93

EGFR: epidermal growth factor receptor.

Supplementary Table 2: Characteristics of patients with lung adenocarcinoma treated with EGFR-TKIs

Characteristics	N	%
**Cases**	158	
median Age, years(range)	55(29-86)	
**Sex**		
male	73	46.2
female	85	53.8
**Smoking status**		
smoker	46	29.1
non-smoker	112	70.9
**PS***		
0-1	138	87.3
2-3	6	3.8
**Histology**		
AC	158	100
**Stage**		
IV	158	100
**Group**		
E746	114	72.2
L747	40	25.3
non-LRE	4	2.5
**EGFR-TKI**		
Erlotinib	82	51.9
Gefitinib	76	48.1

EGFR-TKI: epidermal growth factor receptor-tyrosine kinase inhibitor, AC: adenocarcinoma, PS: performance status; *14 cases with unknown PS.

Supplementary Table 3: Multivariate analysis of factors associated with PFS and OS in 158 patients with advanced lung adenocarcinoma treated with EGFR-TKIs

Variables	PFS		OS	
	P	HR(95%CI)	*P*	HR(95%CI)
**Age(years)**				
<60 versus≥60	0.876	0.971(0.668-1.410)	0.097	1.393(0.941-2.062)
**Sex**				
Male versus Female	0.614	1.218(0.706-1.802)	0.356	1.279(0.759-2.156)
**Group**				
E746	0.525	1	0.723	1
L747	0.266	1.777(0.645-4.897)	0.433	1.501(0.544-4.142)
non-LRE	0.345	1.663(0.578-4.780)	0.523	1.410(0.491-4.053)
**PS**				
0-1 versus 2-3	0.011	3.135(1.302-7.548)	0.454	1.471(0.536-4.039)
**Smoking status**				
non-smoker versus Smoker	0.994	0.998(0.592-1.683)	0.661	1.133(0.647-1.984)
**TKI treatment lines**				
1st versus >2nd	0.234	0.784(0.526-1.170)	0.071	0.666(0.428-1.035)

EGFR-TKI: epidermal growth factor receptor-tyrosine kinase inhibitor; PFS: progression-free survival; OS: overall survival; HR: hazard ratio; CI: confidence interval; AC: adenocarcinoma; ECOG: Eastern Cooperative Oncology Group; PS: performance status.
